# Real-world data combined with studies on Regulatory B Cells for newly diagnosed Multiple Myeloma from a tertiary referral Hospital in South-Western China

**DOI:** 10.7150/jca.53209

**Published:** 2021-03-05

**Authors:** Zhongqing Zou, Tingting Guo, Jian Cui, Wenjiao Tang, Yan Li, Fangfang Wang, Tian Dong, Yunfan Yang, Yan Feng, Matthew Ho, Li Zhang, Ling Pan, Ting Niu

**Affiliations:** 1 Department of Hematology, West China Hospital, Sichuan University, China.; 2 Affiliated Hospital of Chengdu University, Chengdu, Sichuan, China.; 3 Center for Precision Medicine, West China Hospital, Sichuan University, China.; 4 Hematology Research Laboratory, Department of Hematology, West China Hospital, Sichuan University, China.; 5 Department of Internal Medicine, Mayo Clinic, Rochester, Minnesota, United States.

**Keywords:** myeloma, bortezomib, B cell, real world, prognosis

## Abstract

Multiple myeloma (MM) is a heterogeneous disease that remains incurable with significant interpatient variability in outcomes. Regulatory B cells (Bregs) were observed to be involved into specific defects in MM. Here, we provide our risk-adapted approach to newly diagnosed MM (NDMM), combining with the fundamental dysfunction of Bregs. We reported one hundred consecutive patients with NDMM from South-Western China, primarily treated with bortezomib plus dexamethasone with or without a 3^rd^ agent, were enrolled from 2017. Bone marrow aspirates were obtained and flow cytometry (FCM) was used to quantify the percentage of Bregs from the bone marrow. The correlation between Bregs and clinical characters were further analyzed. This study found using bortezomib plus dexamethasone as backbone showed promising efficacy with acceptable tolerability in NDMM. The relatively compromised progression free survival (PFS) points to the essential synergy of bortezomib and lenalidomide here. This study also found that altered proportions of Bregs were closely correlated with treatment efficacy and prognosis in MM. Further understanding of Bregs biology might provide new opportunities to develop immunotherapy, which could prove beneficial in treating MM.

## Introduction

Multiple myeloma (MM) is a heterogeneous disease course and the disease course varies significantly from patient to patient 1. Nonetheless, MM remains an incurable disease and patients inevitably relapse 2. Over the past decade, the introduction of novel agents has greatly improved clinical outcomes. In particular, the clinical use of proteasome inhibitors (e.g., bortezomib and ixazomib) and immunomodulatory drugs (IMiD, e.g., lenalidomide), has paved the way for an exciting era in MM therapy 3. This has created a different but welcomed challenge: one in which clinicians have to decide on the right drugs to use, in the appropriate combination, at the correct time, and in the right sequence for each individual patient based on a multitude of factors, including accessible medical resources, the patients' preferences, and the potential impact on future treatment options. The MM centers have different levels of regional medical resources around the world. Therefore, it is difficult to reach a global uniformity to treat MM, especially in developing countries due to the economic disadvantages 4.

Since 2017, the domestic product of bortezomib or lenalidomide has changed the treatment status of NDMM in China. To be more specific, the application of bortezomib and dexamethasone plus X continues to rage in domestic centers, while the medical insurance agreement has covered bortezomib or lenalidomide for treatment-naïve MM. Bd ± X has become our preferred induction regimen for NDMM. An effort to facilitate updating BD±X to RVD regimen seems to be the next logical step and we also support the concept of the early combination of lenalidomide in high-risk MM 5. It's also worth noting RVD regimen would cost more and currently 6, solid evidence in domestic centers should be needed to support the latter in real world.

One of the most prominent features of MM is immune deficiency which contributes to immune evasion in cancer and predisposes patients to infectious complications 7. Over the last decade, evidence has emerged toward a new regulatory cell type: IL-10-producing B cells, capable of exerting certain immune regulatory functions and therefore termed regulatory B cells (Bregs) with dominant phenotype as CD19+CD24hiCD38hi, in addition to classical regulatory T cells (Tregs) 8-10. Bregs were originally described in autoimmunity animal models to dampen inflammation 11. More recently, Bregs have also been identified in humans 12. Although impaired humoral immunity is a well-established complication of MM 13, there is very limited information available regarding Breg function in MM. MM pathogenesis is closely related to the dysfunctional tumor immune microenvironment and we previously showed that MM patient-derived Bregs reduced the anti-MM activity by NK cells, further supporting the role that Bregs play in MM pathogenesis 14. Despite the presently unanswered questions on Bregs, more studies are necessary to analyze their relevance to disease and therapy in MM.

In this study, we first report the efficacy and safety of Bd ± X in Chinese patients with NDMM. The preliminary exploration of Bregs was also investigated in an attempt to further elucidate the role of Bregs in the pathogenesis of MM. We also discuss possible Breg-directed therapeutic strategies that could potentially reprogram the immune system to harness it against MM.

## Methods

This study was performed at West China Hospital, Chengdu, and approved by our hospital's institutional review board/research ethics board. All subjects provided written informed consent authorizing the use of their data for research purposes.

### Eligibility criteria

This is a real-world, prospective, observational study, we didn't filter any NDMM patients, and all patients were enrolled sequentially in order to investigate the most authentic treatment response. 100 patients from the 35 cities in the Sichuan Province of western China who were over the age of 18 years and newly-diagnosed with symptomatic MM based on the International Myeloma Working Group (IMWG) diagnostic criteria (IMWG 2014) were sequentially enrolled from January 2017 to November 2019. Responses were assessed according to IMWG 2014 uniform criteria. Interphase fluorescence *in situ* hybridization (FISH) analysis of CD138-enriched plasma cells was performed in patients who were able to afford cytogenetic testing. High-risk cytogenetic abnormalities were defined as at least one of the following: del17p, t(4;14), or t(14;16) or a combination of these determined using FISH or karyotype. Treatment-emergent adverse events (TEAEs) that occurred within treatment administration were evaluated using the National Cancer Institute Common Terminology Criteria for Adverse Events (NCI CTCAE version 4). Quantitative immunoglobulin levels were analyzed both at diagnosis and post-treatment and immunoparesis was defined as one or more uninvolved immunoglobulins below the lower limits of normal. Data collection ceased 31 December, 2019, after which data was analyzed.

### Initial therapy

**Figure 1:**
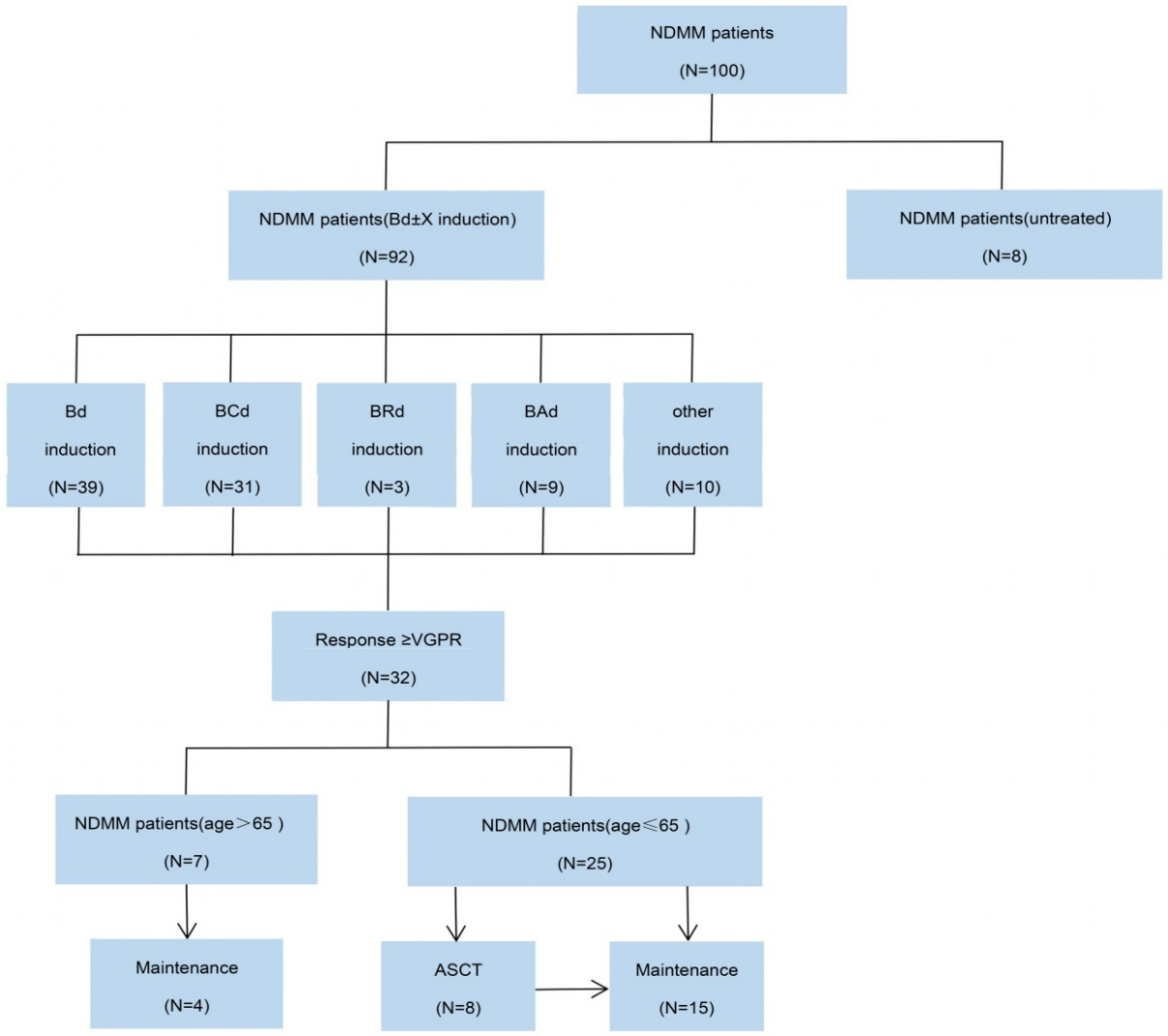
CONSORT diagram. NDMM, newly diagnosed multiple myeloma; Bd, bortezomib plus dexamethasone; BCd, bortezomib, cyclophosphamide and dexamethasone; BRd, bortezomib, lenalidomide and dexamethasone; Bad, bortezomib, pegylated liposomal doxorubicin and dexamethasone; VGPR, very good partial response; ASCT, autologous stem-cell transplantation.

In treatment decision making, BD regimen has become a backbone to which several other agents have been integrated. A BD-based combination (e.g., bortezomib-dexamethasone-cyclophosphamide) is called as BD±X regimen. Bd ± X was administered every 4 weeks or 1 natural month 1.3 mg/m^2^ of bortezomib was administered subcutaneously weekly on days 1, 8, 15 and 22, and 20 mg/m^2^.week of dexamethasone was administered on the day of and the day after bortezomib administration. Subcutaneous bortezomib was chosen due to better neurotoxicity profile, ease of administration in the outpatient setting, and patient comfort. The 3^rd^ agent termed as X was added or omitted under certain conditions (Figure 1). Specifically, for the patients with good tolerability to Bd regimen, specific indications for initiation of the other therapy for MM existed. For patients with renal impairment and/or amyloidosis attributable to MM, cyclophosphamide was in combination with Bd regimens using a 300 mg/m^2^ dosing on days 1, 8, 15 and 22 per cycle. For patients with extramedullary involvement received 40 mg of pegylated liposomal doxorubicin (in combination with Bd backbone) on days 1 of each cycle. Following induction with Bd ± X, stem cell harvest is all recommended for patients who met eligibility criteria (i.e., ≤ 65 years and ≥ Very good partial response and in first remission). Upfront autologous stem cell transplantation (ASCT) was performed for patients from whose consent was obtained. All patients were given routine maintenance with either lenalidomide or bortezomib, given both patients' intent and risk stratification.

Additionally, patients on an emergent basis with hyperviscosity related symptoms were treated with dexamethasone and cyclophosphamide and/or therapeutic plasma exchange as an important adjunctive to facilitate rapid removal of circulating M proteins. Isolation of bone marrow mononuclear cells (BMMNCs) Heparinized bone marrow was obtained from patients with NDMM prior to treatment. Plasma was separated from whole-blood cells by centrifugation at 800 g for 20 min. The layer containing the BM mononuclear cells was isolated and added to an equal volume of lymphocyte isolation buffer (Hao yang Bio-Sciences, Tianjin, China) for lymphocyte isolation by centrifugation. The lymphocyte layer was then gently transferred into a new tube and washed with 5 mL of serum-free RPMI 1640 medium. Lymphocytes were pellet by centrifugation at 2000 RPM for 7 min and supernatant was discarded. Red blood cells (RBCs) were lysed by adding 1ml of RBC lysate for 3 min and lymphocytes were washed with 5 mL PBS, followed by centrifugation at 2000 RPM. Lymphocyte pellet was then resuspended in RPMI 1640 with 10% fetal bovine serum (FBS) and total number of cells was counted following trypan blue discrimination and the volume of media was adjusted to keep cell density at 2×10^6^/mL for further use.

### Determination of BM-derived CD19+CD24hiCD38hi Bregs

BMMNCs were washed twice in PBS. After discarding the supernatant, BMMNCs were incubated with antibodies against CD38 (PE-cy7), CD19 (FITC), and CD24 (APC) (BioLegend ) for 15 min. Excess (unbound) antibodies were removed by washing with PBS and cells were re-suspended in 0.2 mL PBS for flow cytometry detection (Beckman).

### Statistical analyses

Responses were categorized based on IMWG criteria and, for each response category, a 2-sided 95% exact confidence interval (CI) was calculated. Overall response rate (ORR) was analyzed for all treated patients and defined as the proportion of patients with stringent complete response (sCR), complete response (CR), very good partial response (VGPR), or PR. Distributions of PFS and overall survival (OS) were calculated using the Kaplan-Meier method, and differences among survival curves were analyzed by the log-rank test. PFS was defined as the time from diagnosis to progression or death from any cause. OS was defined as the time from diagnosis to death from any cause. Significant risk factors for both PFS and OS in the univariable analysis were included in a multivariable Cox proportional hazards regression model. The χ^2^ test and the Fisher exact test were used to test for the independence of categories. The t test was used for comparing means between 2 groups. Correlation analysis was performed by the Spearman test. Statistical significance was defined when *P*<0.05. SPSS 23.0 software was used to process all collected data.

## Results

### Characteristics of the patients

A total of 100 patients were consecutively enrolled in this retrospective study and their baseline characteristics are presented in Table 1. Of the 100 patients with NDMM, the ratio of male to female was 1.17 and the median age was 61 years old. Sixty-one patients (61%) had elevated serum LDH, while 19% had impaired renal function at diagnosis. The most common involved types of heavy chains were IgG and IgA. The distribution of the involved light chain as kappa to lambda was 1.06. Of the 69 patients with available cytogenetic data at screening, 20.29% (14 cases) had high-risk cytogenetic abnormalities and 42.03% (29 cases) had a gene amplification at chromosome 1q21 (amp1q21). In total, 89.1% of the patients had received bortezomib and 12.0% had received lenalidomide. The most commonly used induction regimens were Bd (45%) and bortezomib, cyclophosphamide, and dexamethasone (Bd+C) (41%), while only 4 patients received RVd (4%). The median (range) number of inductive treatment cycles was 8 (3-12). Up to the cutoff, 8 patients underwent ASCT after induction. Of the 100 enrolled patients 23% discontinued follow-up: 16% because of withdrawal of consent and 7% because of death.

### Efficacy and Survival

The evaluable response analysis set included 80 patients who received ≥1 cycle of any component of study treatment, ORR was 93.8% in all treated patients. Of the 41 patients who completed the induction treatment (Table 2), 39.02% achieved a best response of CR or better and 78.05% achieved VGPR or better. In an analysis of patients by baseline cytogenetic risk, 28.57% of the patients with high-risk features achieved CR compared with 52.38% for standard-risk patients.

**Figure 2:**
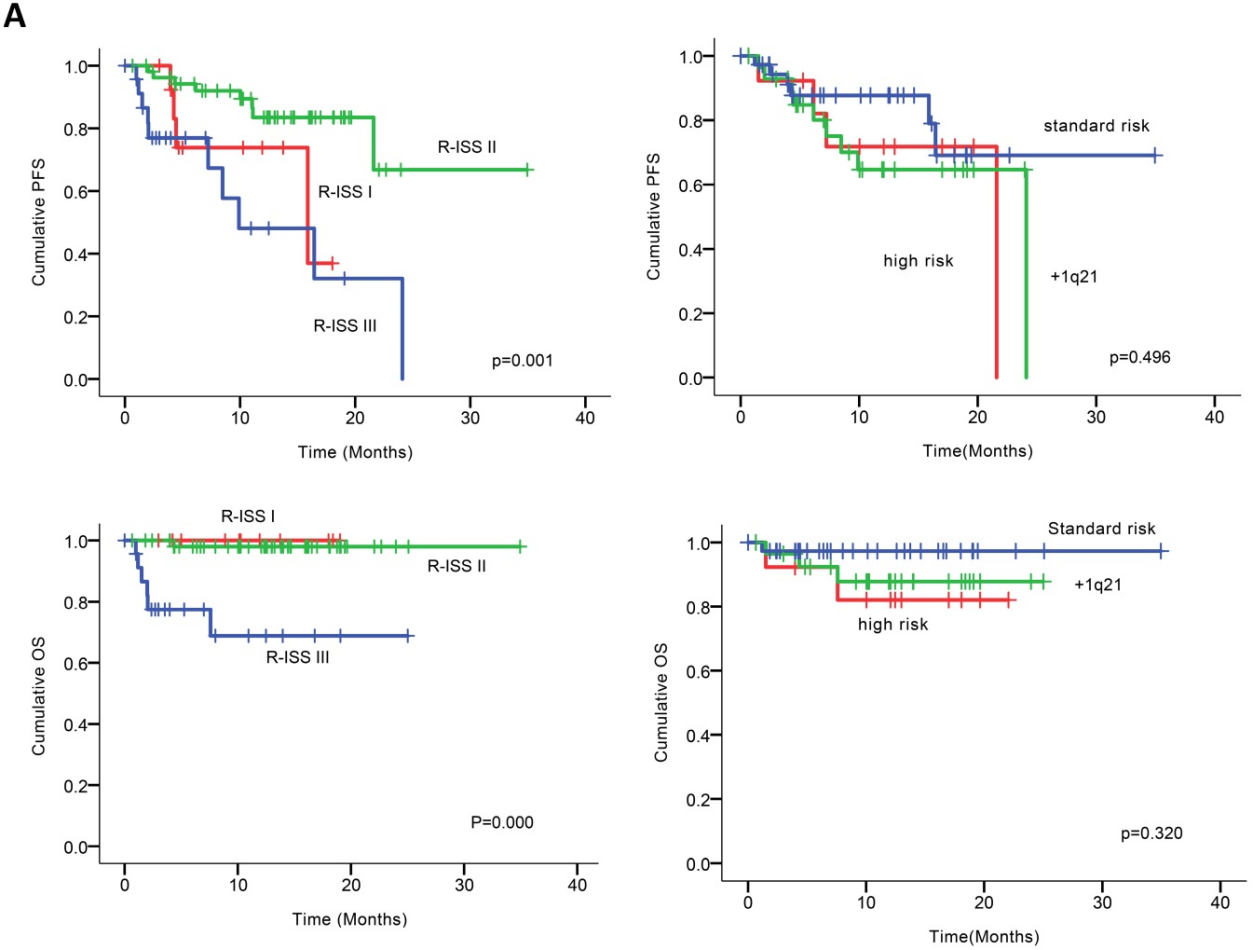

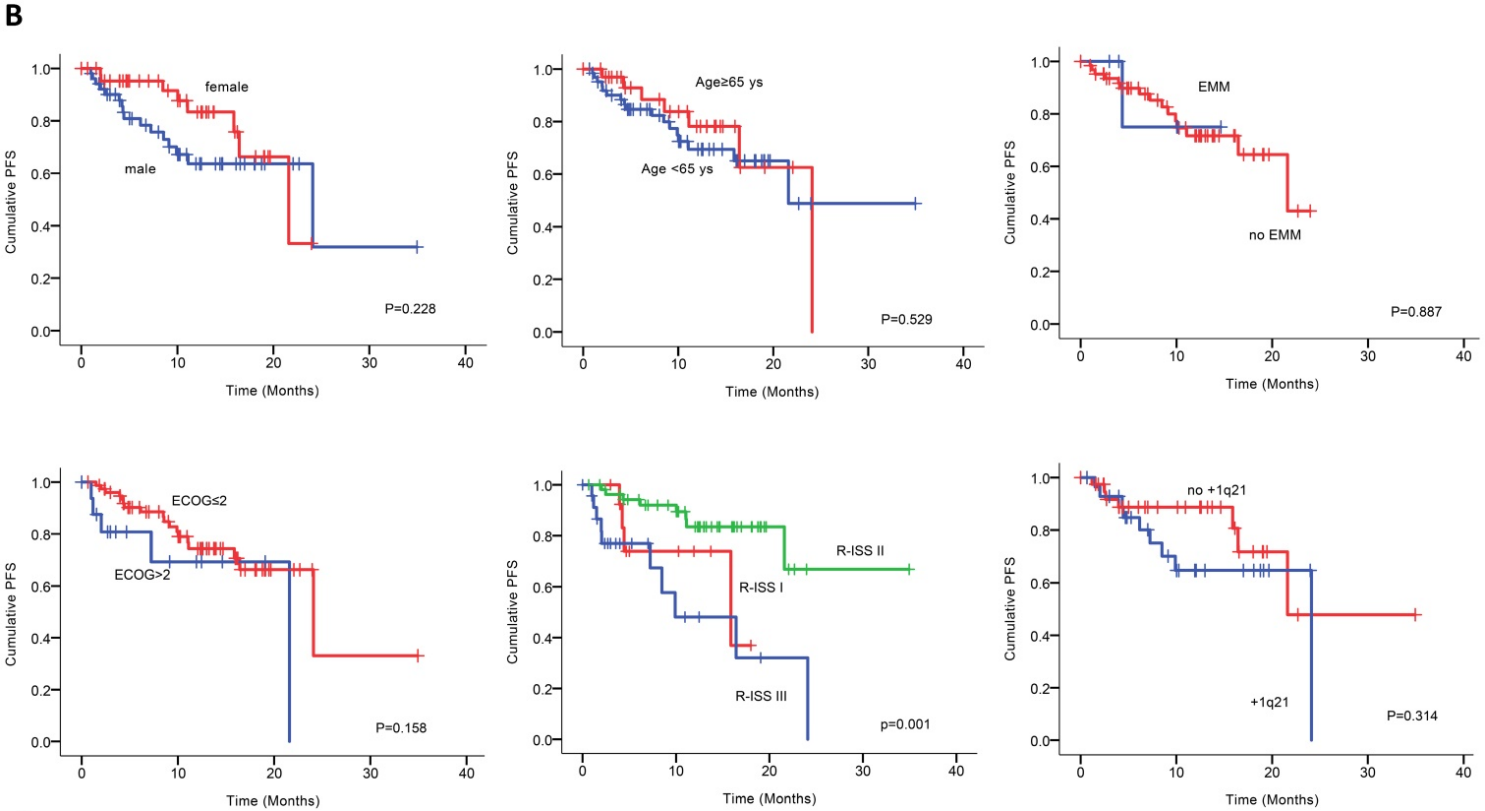

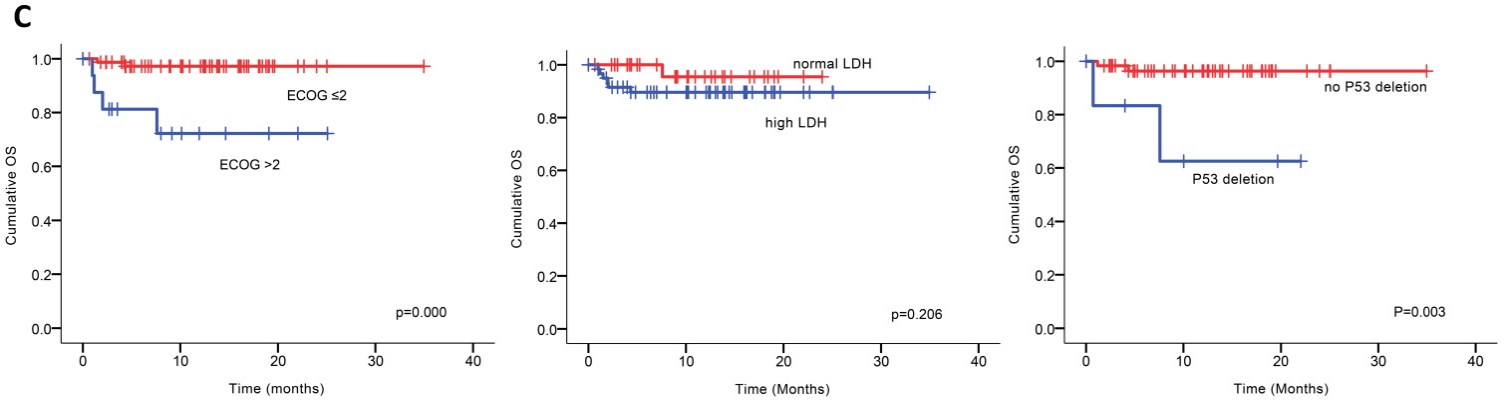
** (A)** PFS and OS among patients with newly diagnosed MM according to RISS, genetic abnormalities. **(B)** Univariable significant risk factors were Men, Age <65, extramedullary multiple myeloma (EMM) of initial diagnosis, high ECOG score, R-ISS score and 1q 21 amplification for PFS.2. **(C)** Univariable significant risk factors were high ECOG score, LDH and P53 deletion for OS.

At the clinical cutoff, the median follow-up was 10.26 (0.00-34.95) months (Figure 2), while the median time to progression was 24.10 months (95%CI 16.19-32.01). During follow-up, the cumulative probability of progression was 12.64% at 6 months, 27.64% at 1 year, 34.50% at 18 months, and 72.77% at 2 years; approximately doubling every 6 months. In an exploratory analysis of PFS based on cytogenetic risk classification at baseline, the 12-month PFS rate was 87.7% in patients with standard risk (n = 38), 71.8% and 64.7% in those with high-risk status (n = 14) and those with amp1q21 (n = 29); 18-month PFS rates were 69.1%, 68.8% and 64.5%, respectively. However, the median PFS was not reached in standard risk patients while 21.6 and 24.1 months in the high-risk patients and those with amp1q21, respectively. Median OS had not been reached in the overall treated population at the time of analysis, while the estimated 24-month OS rate was 91.9%. Likewise, lower OS rates were found in the high-risk patients and those with amp1q21 compared to standard risk patients.

Univariable analysis of risk factors for poor performance was significant for gender, age, extramedullary infiltration, ECOG scores, R-ISS scores and amp1q21 for PFS, and ECOG scores, elevated LDH and P53 deletion for OS (Figure 2). Multivariable analysis revealed that RISS stage III [*P*=0.003, HR 8.117 (95%CI, 2.004-32.880)] represented an independent poor prognostic factor for PFS and deletion of P53 [*P*=0.031, HR13.952 (95%CI, 1.275-152.655)] for OS in NDMM.

### Safety and toxicity

The evaluable safety analysis set included 84 patients who received ≥1 cycle of any component of study treatment. The most frequent all-grade TEAEs reported were respiratory tract infection (19.05%), herpes zoster (HZ) infection (10.71%), thrombocytopenia (4.76%), diarrhea (3.57%) and peripheral neurotoxicity (2.38%). The most common grade 3/4 TEAEs requiring hospitalization were pneumonia observed in 10 patients (11.9%). Among them, 7 cases were associated with anti-MM treatment delays which may limit efficacy. To maximize dose delivery and reduce toxicity, we modified the original schedule by reducing dose or deleting the 3^rd^ agent of X for the left 3 patients to avoid therapeutic discontinuation.

### Altered bone marrow B-cell compartment and percentages of regulatory B cells in patients with NDMM

**Figure 3:**
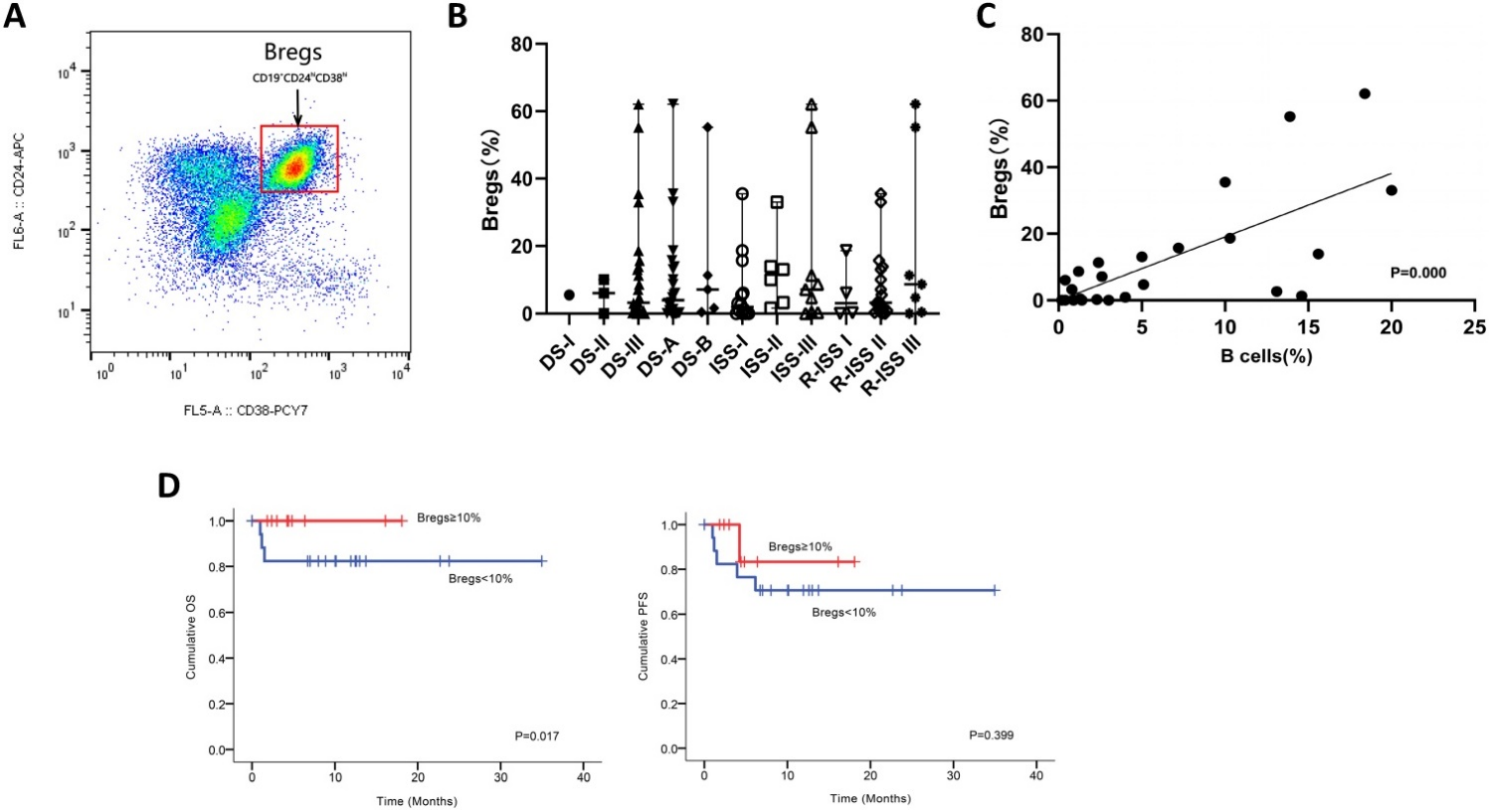
Phenotypic analysis and frequencies of bone marrow B-cell subpopulations in patients with NDMM. **(A)** Bone marrow from patients were stained with CD19, CD24, and CD38. CD24 and CD38 expression patterns of CD19 lymphocyte gated cells are shown in the representative dot plots of MM patients. The gating strategy for analysis of the CD24hiCD38hi B cells is indicated as Bregs. **(B)** Frequencies of CD24hiCD38hi of CD19 subset in MM patients at different stages are indicated. **(C)** Using the Spearman test, we found a significantly positive correlation between CD19+CD24hiCD38hi Bregs' percentages within CD19+ B-cell ratios and CD19+ B-cell ratios within BMMNCs. The symbols represent individual samples, the horizontal bars represent the mean. Significant differences are indicated as follows: *P <0.05. **(D)** Kaplan Meier overall survival and progression free survival curves by frequencies of Bregs.

We next utilized FCM analysis to characterize Bregs in 29 patients with NDMM (Figure 3A). Our preliminary data showed the frequencies of CD19+CD24hiCD38hi Bregs within CD19+ B cells significantly varied among different disease subtypes of plasma cell neoplasms; and the patients at early stage had significantly higher levels of Bregs' ratios than at late stage. Namely, when compared to MM, patients with MGUS had significantly higher percentages of CD19+CD24hiCD38hi Bregs 15. Accordingly, in this study, we first stratified patients based on DS, ISS and RISS, which are clinical indicators disease severity. However, we did not detect any significant difference in Breg frequencies among the different stages of NDMM patients analyzed, regardless of B cell ratios. Immunoparesis, defined as below normal levels of at least one uninvolved immunoglobulin, has been reported to be associated with an unfavorable prognosis in NDMM 16, 17. Univariable analysis did not show any correlation between Breg' frequency and qualitative nor quantitative degree of immunoparesis in our study cohort.

Notably, there was a statistically significant positive correlation [*P*=0.000] between the proportion of B cells and the proportion of Bregs in NDMM (i.e., patients with a low proportion of B cells also had a low proportion of Bregs) (Figure 3C). Furthermore, patients with < 10% (0-62.1%) Bregs (within CD19+ B-cell compartment) had significantly worse OS and also presenting a tendency of worse PFS (Figure 3D).

## Discussion

MM has benefited from several practice-altering advances in recent years bolstered, in a large part, by the introduction of a wide array of novel therapies. However, this has also led to increasingly complex therapies that pose a challenge in terms of deciding the optimal combination of drugs, right timing, and correct sequence. Notably, the majority of our cases with NDMM presented elevated LDH, directly defined as RISS 3. Our goals of therapy were to achieve VGPR without compromising the quality of life in these patients. As such, minimal residual disease (MRD) testing is not routine practice here. Results from this real-world study show that combination therapy with Bd ± X, at a lower treatment intensity than RVD, has comparable efficacy to CyBorD in clinical trials and off-clinical trials at Mayo prior to SMART 3.0 18-21. Bd ± X regimen is well-tolerated and absence of thalidomide may be reasonable for reducing neuropathy under exposure to bortezomib. At the time of our study, upfront ASCT for NDMM was not a routine practice although its first-line role as consolidation has been already determined by solid medical evidence. This required us to conduct patient education on ASCT and scale up transplantation centers. The fact that median OS was not reached in our study is reflective of MM as a relatively indolent malignancy. Additionally, our study shows that Bd ± X was not able to overcome the deleterious impact of adverse cytogenetics (e.g., TP53 deletion) on OS.

The frequency of chromosomal aberrations in our cohort was similar to previously reported studies 22. Consistent with previously published reports on Asian MM patients, amp1q21 is one of the most frequently recurring chromosomal aberrations in our cohort, with approximately 50% of our patients haboring this amp1q21 23. The amplification of 1q21 is an adverse prognostic factor in patients with multiple myeloma in Chinese population 24, 25. We also showed that amp1q21 was prone to predict poor OS in NDMM, with similar outcomes to patients with high-risk cytogenetics and in keeping with other evidence establishing its association with increased risk of progression and worse survival. Amp1q21 was not found to be an independent adverse prognostic factor in this study, possibly due to the limited sample size. Additionally, in the last decade, the cellular and molecular mechanisms of amp1q21 in plasma-cell dyscrasias are poorly understood. For example, whether amp1q21 is a driver or passenger in clinical course of MM remains to be seen. Studies to determine the precise role of the involved candidate genes within the region of chromosome 1q21 are ongoing 26, 27, and notably, amp1q21 has recently been classified as a high-risk cytogenetic abnormality by the Mayo group, and has been linked with poor prognosis in MM according to NCCN 2020 28. Therefore, a comprehensive study to clarify the direct implications and prognostic significance of amp1q21 in MM is needed. On the other hand, amp1q21 was reported as an independent factor predicting OS in the context of treatment with lenalidomide and dexamethasone in recurrent MM 29, and might be an important predictor of response to thalidomide in patients with NDMM 22. Specifically, patients with amp1q21 may be not benefited from thalidomide 22. Hence, we recommend the early use of novel agents such as lenalidomide especially for NDMM patients with amp1q21 and/or other high-risk phenotype, consistent with recurrent recommendations that RVD is the preferred therapy for NDMM.

Bd ± X does not maximize responses with similar ORR but relatively shorter PFS, which can further be consolidated and improved by RVD regimen, especially for MM with high risk cytogenetics, although the ORR in our study was comparable to that of RVd in clinical trials. The median PFS in our NDMM treated by Bd-based induction is 24.10 months, obviously inferior to the 65 months in patients treated with RVD regimen very recently reported in real world study 30. The country has many regional agreements of domestic medical insurance. In China, since 2020, it has already begun to make an important agreement to cover both bortezomib and lenalidomide for NDMM, which has a profound effect on eliminating therapeutic costs for MM patients here. There is an increasing need for the addition of lenalidomide to Bd especially for high risk NDMM.

In this study, Bd ± X was safe and well-tolerated. The rate of neuropathy was low, to some extent, resulted from rarely usage of thalidomide here. Bortezomib and/or dexamethasone have been associated with HZ and although approximately 10% of our treated patients had HZ reactivation, their HZ was manageable and resolved without complications upon dexamethasone and bortezomib dose adjustment.

MM is associated with dysfunctional humoral and cell mediated immunity 31. Importantly, current immunotherapeutic interventions such as daratumumab and Chimeric Antigen Receptor T-cell Immunotherapy (CAR-T), besides providing deep clinical responses, may also improve immune cell function with impact on long term outcomes^32^. Most studies around the immunosuppressive BM microenvironment focus on T regulatory cells (Tregs), myeloid derived suppressors cells (MDSCs), and plasmacytoid dendritic cells (pDCs) 33, 34. Hence, the role of B cells in modulating the immune response in the setting of both solid tumors and lymphoid malignancies including MM is less well understood. Over the past decade however, emerging evidence demonstrates that Bregs are crucial in the maintenance of immune tolerance and the suppression of inflammation within BM milieu 14, 35-37. However, the role that CD19+CD24hiCD38hi Bregs play in MM is currently unknown. Our observation that Breg proportion <10% relative to the B-cell pool predicts worse survival needs to be confirmed in larger prospective studies. We also found a positive correlation between CD19+CD24hiCD38hi Breg percentages and B cell percentages. Thus, higher frequencies of Bregs may be associated with positive outcomes in MM. One hypothesis is that increased frequency of Bregs' subpopulation in MM patients can represent a lower tumor burden and a relatively higher immune reservoir, although direct proof of this will have to await the isolation and characterization of this subset in patients with early-versus-late stages in NDMM based on a larger sample size. The use of B cell depletion therapy (i.e., rituximab) has shown some success in the treatment of autoimmune diseases and B cell lymphoma 38, 39. Similarly, it would be advantageous to immune restoration for NDMM if selectively depleting immunosuppressive Bregs by specific antibodies, such as daratumumab. Improved understanding of Breg induction and function in tumor immunology could result in Breg-targeted therapies that enhance antitumor immunity. Collectively, this suggests that new therapies targeting Breg' activity could be a promising approach to treat MM, especially in the maintenance.

Our study is limited by the small sample size, and a relatively short median follow-up period of only 10.26 months. Longer follow-up is required to assess the efficacy of Bd ± X regimen for NDMM. It should be stressed that future studies with larger and more focused patient groups are needed to confirm these findings of Bregs and explore the possible effect of the drugs on the Breg compartment. Because of the retrospective nature of the current study, the potential clinical importance and utility of the findings reported here will require confirmation with larger prospective and longitudinal studies.

## Conclusions

In summary, our findings complement the results of our current condition on NDMM in Southwestern China, in which Bd±X induced a high ORR, a relatively satisfying survival and controllable adverse toxicity. Patients with rapid relapses or who have poor survival, highlighting that medical insurance covering more novel therapies and treatment combinations are urgently needed in these patients. Due to limited PFS, patients with relapsed MM who previously benefited from Bd±X also represent a growing population with a high unmet need for RVD, a more potent option than Bd±X. In the present study, addition of Bregs to Bd±X resulted in considerable data regarding the prognostic and therapeutic implications of Bregs in NDMM, requiring further confirmation accompanying with enlargement of sample size and exploration of biologically mechanisms. Recognizing the paucity of data, we advocate participation in translational medicine research on Bregs in the era of RVD.

## Figures and Tables

**Table 1:** Patient demographics and clinical characteristics

Characteristics	Cases (%)
**Sex**	
Male	54 (54.0%)
Female	46 (46.0%)
**Age (years)**	61 (26-81)
<65	64 (64.0%)
≥65	36 (36.0%)
**ECOG score**	
≤2	79 (79.0%)
>2	17 (17.0%)
miss	4 (4.0%)
**LDH**	
≤220	39 (39.0%)
220	61 (61.0%)
**Heavy chain**	
IgG	57 (57.0%)
IgA	22 (22.0%)
IgD	3 (3.0%)
IgM and IgG	1 (1.0%)
Light chain	15 (15.0%)
miss	2 (2.0%)
**Light chain**	
κ	50 (50.0%)
λ	47 (47.0%)
miss	3 (3.0%)
**Durie-Salmon stage**	
DS-I	5 (5.0%)
DS-II	16 (16.0%)
DS-III	78 (78.0%)
miss	1 (1.0%)
DS-A	81 (81.0%)
DS-B	19 (19.0%)
**International staging system**	
ISS-I	43 (43.0%)
ISS-II	24 (24.0%)
ISS-III	32 (32.0%)
miss	1 (1.0%)
**Revised International staging system**	
R-ISS I	14 (14.0%)
R-ISS II	55 (55.0%)
R-ISS III	24 (24.0%)
miss	7 (7.0%)

ECOG: Eastern Cooperative Oncology Group.

**Table 2:** Response rates to bortezomid and dexamethasone based induction therapy in NDMM

Response	ALL (N=41)	Standard risk (N=21)	High disk (N=7)	+1q21 (N=9)
≥CR	16 (39.02%)	11 (52.38%)	2 (28.57%)	2 (22.22%)
≥VGPR	32 (78.05%)	17 (80.95%)	7 (100%)	6 (66.67%)
≥PR	38 (92.68%)	20 (95.24%)	7 (100%)	8 (88.89%)
sCR	6 (14.63%)	6 (28.57%)	0 (0%)	0 (0%)
CR	10 (24.39%)	5 (23.81%)	2 (28.57%)	2 (22.22%)
VGPR	16 (39.02%)	6 (28.57%)	5 (71.43%)	4 (44.44%)
PR	6 (14.63%)	3 (14.29%)	0 (0%)	2 (22.22%)
SD	0 (0%)	0 (0%)	0 (0%)	0 (0%)
PD	3 (7.32%)	1 (4.76%)	0 (0%)	1 (11.11%)

CR: complete response; VGPR: very good partial response; PR: partial response; sCR: Stringent complete response; SD: stable disease; PD: progressive disease.
