# Real-world data combined with studies on Regulatory B Cells for newly diagnosed Multiple Myeloma from a tertiary referral Hospital in South-Western China: Erratum

**DOI:** 10.7150/jca.79561

**Published:** 2022-11-03

**Authors:** Zhongqing Zou, Tingting Guo, Jian Cui, Wenjiao Tang, Yan Li, Fangfang Wang, Tian Dong, Yunfan Yang, Yan Feng, Matthew Ho, Li Zhang, Ling Pan, Ting Niu

**Affiliations:** 1 Department of Hematology, West China Hospital, Sichuan University, China.; 2 Affiliated Hospital of Chengdu University, Chengdu, Sichuan, China.; 3 Center for Precision Medicine, West China Hospital, Sichuan University, China.; 4 Hematology Research Laboratory, Department of Hematology, West China Hospital, Sichuan University, China.; 5 Department of Internal Medicine, Mayo Clinic, Rochester, Minnesota, United States.

The authors recently noticed a mistake in the initially published version of our article. When combining different images into Figure 2, an identical image of R-ISS, shown at upper left in Figure 2A, was selected as the middle image at the lower line in Figure 2B. The corrected Figure 2 and the corresponding figure legend are provided below. The error does not alter any results and conclusions of this study. All authors agree to the erratum and regret any inconvenience that it may have caused.

## Figures and Tables

**Figure 2:**
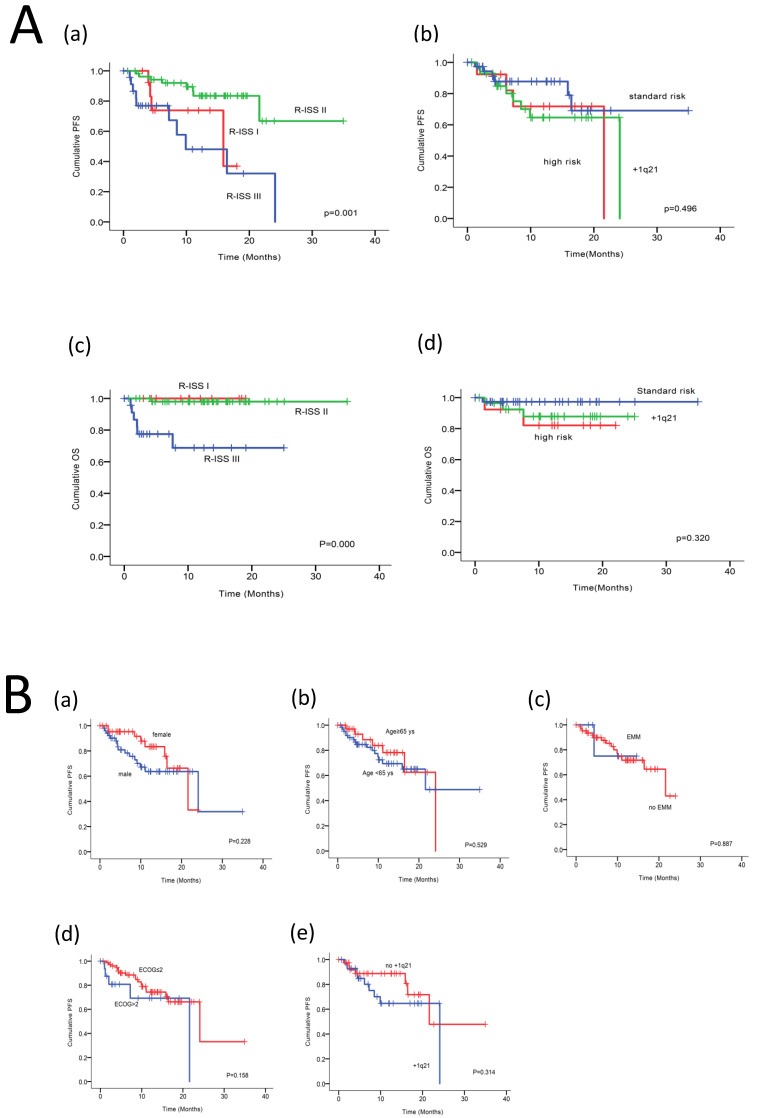

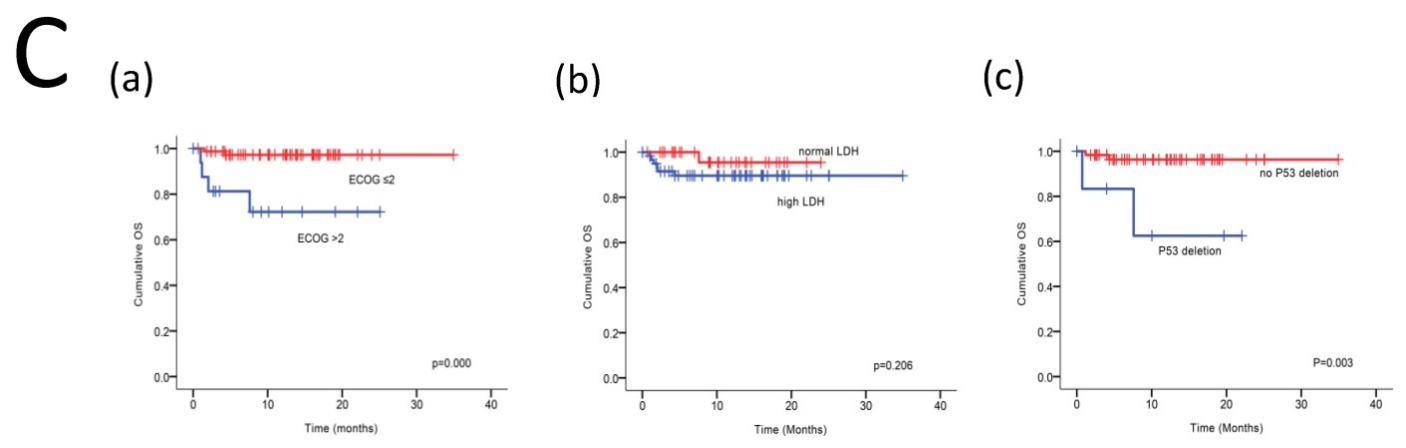
** (A)** PFS and OS among patients with newly diagnosed MM according to RISS(a, c), genetic abnormalities(b, d). **(B)** Univariable significant risk factors were Men(a), Age <65(b), extramedullary multiple myeloma (EMM) of initial diagnosis(c), high ECOG score(d), R-ISS score(Figure 2A-a) and +1q 21 (e) for PFS. **(C)** Univariable significant risk factors were high ECOG score(a), LDH(b) and P53 deletion (c) for OS.

